# Patient-Specific Finite Element Models of Posterior Pedicle Screw Fixation: Effect of Screw’s Size and Geometry

**DOI:** 10.3389/fbioe.2021.643154

**Published:** 2021-03-10

**Authors:** Marco Sensale, Tanguy Vendeuvre, Christoph Schilling, Thomas Grupp, Michel Rochette, Enrico Dall’Ara

**Affiliations:** ^1^Ansys France, Lyon, France; ^2^Department of Oncology and Metabolism, Mellanby Centre for Musculoskeletal Research, University of Sheffield, Sheffield, United Kingdom; ^3^INSIGNEO Institute for in Silico Medicine, University of Sheffield, Sheffield, United Kingdom; ^4^Spine and Neuromodulation Functional Unit, Poitiers University Hospital, Poitiers, France; ^5^PRISMATICS Lab (Predictive Research in Spine/Neuromodulation Management and Thoracic Innovation/Cardiac Surgery), Poitiers University Hospital, Poitiers, France; ^6^Aesculap AG, Research and Development, Tuttlingen, Germany; ^7^Department of Orthopaedic Surgery, Physical Medicine and Rehabilitation, Ludwig Maximilians University of Munich, Munich, Germany

**Keywords:** spine, spinal fractures, posterior fixation, finite element, sensitivity analysis

## Abstract

Pedicle screw fixation is extensively performed to treat spine injuries or diseases and it is common for thoracolumbar fractures. Post-operative complications may arise from this surgery leading to back pain or revisions. Finite element (FE) models could be used to predict the outcomes of surgeries but should be verified when both simplified and realistic designs of screws are used. The aim of this study was to generate patient-specific Computed Tomography (CT)-based FE models of human vertebrae with two pedicle screws, verify the models, and use them to evaluate the effect of the screws’ size and geometry on the mechanical properties of the screws-vertebra structure. FE models of the lumbar vertebra implanted with two pedicle screws were created from anonymized CT-scans of three patients. Compressive loads were applied to the head of the screws. The mesh size was optimized for realistic and simplified geometry of the screws with a mesh refinement study. Finally, the optimal mesh size was used to evaluate the sensitivity of the model to changes in screw’s size (diameter and length) and geometry (realistic or simplified). For both simplified and realistic models, element sizes of 0.6 mm in the screw and 1.0 mm in the bone allowed to obtain relative differences of approximately 5% or lower. Changes in screw’s length resulted in 4–10% differences in maximum deflection, 1–6% differences in peak stress in the screws, 10–22% differences in mean strain in the bone around the screw; changes in screw’s diameter resulted in 28–36% differences in maximum deflection, 6–27% differences in peak stress in the screws, and 30–47% differences in mean strain in the bone around the screw. The maximum deflection predicted with realistic or simplified screws correlated very well (*R*^2^ = 0.99). The peak stress in screws with realistic or simplified design correlated well (*R*^2^ = 0.82) but simplified models underestimated the peak stress. In conclusion, the results showed that the diameter of the screw has a major role on the mechanics of the screw-vertebral structure for each patient. Simplified screws can be used to estimate the mechanical properties of the implanted vertebrae, but the systematic underestimation of the peak stress should be considered when interpreting the results from the FE analyses.

## Introduction

In the lumbar spine, pedicle screw fixation is the most widespread technique to achieve spinal fusion and stabilization (Verma et al., 2016). In 2008, approximately 415,000 spinal fusion surgeries were performed in the United States alone (Rajaee et al., 2012). The global pedicle screw system market has been predicted to increase of about 32% from 2018 to 2025 as reported by Fior Markets (Fior Markets, 2020). Pedicle screw fixation is the standard surgical procedure to treat different diseases of the spine, in particular, it is common for thoracolumbar fractures.

Despite the extensive use of pedicle screws in the current clinical practice, screw loosening and screw breakage are recurring mechanical complications of spinal fixation that can bring to a revision surgery in about 6% of cases (Prud’homme et al., 2015; Bredow et al., 2016). For this reason, surgery-related parameters should be optimized to improve the outcomes of this surgery. While surgeons decide the optimal size, insertion point and orientation of screws based on anatomical measurements on CT-images, finite element (FE) models are efficient tools to mechanically assess the stability of different configurations of the instrumented spine under different loading conditions. FE models of the vertebra should take into account different parameters related to the bone geometry, bone tissue heterogeneity, different boundary conditions, and before clinical applications they should be verified and validated [see for example (Assessing Credibility of Computational Modeling through Verification and Validation: Application to Medical Devices–ASME)]. Many studies assessed the optimal fixation to treat a burst fracture by simulating with FE models a system composed of three vertebrae and two intervertebral discs implanted with different configurations of rod and screws (e.g., monolateral vs. bilateral, short segment vs. long segment) (Li et al., 2014; Elmasry et al., 2017; Su et al., 2018; Wang et al., 2019). Other studies focused on the vertebra-screws interactions and proposed FE models validated with experimental measures: FE models were found to be good predictors of pull-out strength and stiffness obtained by experimental tests better than apparent density estimated from CT images (Abbeele et al., 2018; Chevalier et al., 2018; Widmer et al., 2020). The screw size and other insertion-related parameters have been tested with linear FE models (Qi et al., 2011; Newcomb et al., 2017), with non-linear FE models (material non-linearities, contact mechanics) (Chen et al., 2003; Bianco et al., 2017, 2019; Molinari et al., 2021), or assuming the bone as heterogeneous material with elastic properties driven by the local bone mineral density (BMD) (Matsukawa et al., 2016, 2020; Biswas et al., 2019; Molinari et al., 2021). In most cases a realistic screw geometry was used and only in a few studies the simplified geometry of the screw was modeled (Li et al., 2014; Su et al., 2018). The usage of simplified screws would enable the optimization and automation of the modeling procedure to evaluate vertebral and screws properties, if used in combination with morphing and reduced model order techniques (Campbell and Petrella, 2016). Although FE models of the instrumented spine are often proposed as tools for planning pedicle screw fixations to predict the optimal screw size and orientation for a given patient, little is known about the capability of predicting the biomechanical properties of the screw and of the vertebra if simplified or realistic screws are used. In particular, to the authors’ knowledge, a comprehensive assessment of the effect of the mesh size and the sensitivity of the models to the screw size and geometry, in terms of stress in the screw, strain in the heterogeneous bone, and deflection of the screw within the bone, has not been reported in the literature yet. This gap in the literature makes it difficult to compare the outcomes from different studies and understanding the potential of the FE models in evaluating the biomechanics of the implanted vertebrae.

The aim of this study was to verify and evaluate the sensitivity of subject specific FE models of the vertebra with two pedicle screws for different sizes of the implant and in case of realistic and simplified geometry of the screw.

## Materials and Methods

**FIGURE 1:**
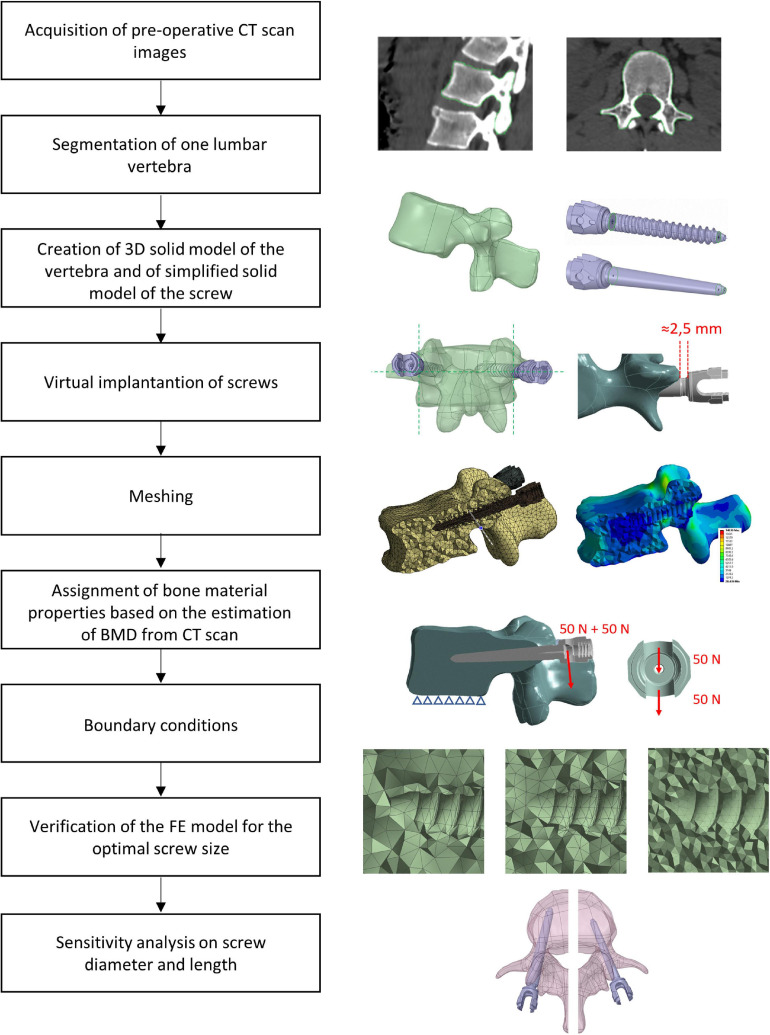
Workflow used to generate, verify, and test the sensitivity of heterogeneous FE models of one vertebra implanted with two pedicle screws.

Anonymized CT-scans of the thoracolumbar spine of three patients were collected. One vertebra per patient was segmented, converted to a FE model, virtually implanted with pedicle screws, and vertical loads were applied to the head of the screws, perpendicular to their axis. A mesh refinement study for realistic or simplified geometry of the screws was performed to choose the optimal mesh size that was used to evaluate the sensitivity of the model to changes in screw’s size (diameter and length) and geometry (realistic or simplified). An overview of the study is presented in Figure 1.

### Imaging and Image Processing

Three anonymized clinical pre-operative CT-scans of the thoracolumbar spine of three patients were analyzed. The scans were previously acquired at the University Hospital Centre (CHU) of Poitiers (France) and transferred only after anonymization (CHU86-RECH-R2020-02-01). These patients were treated with a posterior pedicle screw fixation for different reasons: two patients reported a vertebral fracture at L1 (Patients #1 and #3), one patient had osteoarthritis (Patients #2). The scanning parameters are reported in Table 1. In order to simplify the sensitivity study one vertebra with similar size was selected from each patient (L2, L3, and L4 for Patient #1, Patient #2, and Patient #3, respectively). The relative difference in the mean CT based BMD in the vertebral bodies was 21% between Patient #1 and Patient #2 and −24% between Patient #1 and Patient #3.

**TABLE 1:** Parameters of acquisition of CT-scan images for the three patients.

Scanning parameters	Patient #1	Patient #2	Patient #3
Voltage (kV)	120	135	135
Current (mA)	181	200	273
Exposure (s)	1.38	0.5	0.5
In plane pixel size (mm^2^)	0.98 × 0.98	0.88 × 0.88	0.68 × 0.68
Slice thickness (mm)	1.25	1.0	1.0
Model, manufacturer	Optima CT540, GE Healthcare, United States	Aquilion, Toshiba, JP	Aquilion, Toshiba, JP

The pedicle widths and the distances between the approximated insertion points and the anterior wall of the vertebral body were measured in a cross-section corresponding to the approximated insertion points and including the longitudinal axes of the screws. From these measurements, and based on the advice of an experienced surgeon, it was concluded that the size of the vertebrae was ideal for the insertion of pedicle screws with diameter (D) equal to 6.5 mm and length (L) equal to 45 mm. The shape of the vertebrae was reconstructed by manual image segmentation of the CT cross-sections (3D Slicer, v4.10.1) (Fedorov et al., 2012). The resulting mask was smoothed with a Laplacian smoothing. The number of iterations was adjusted in order to preserve the geometric features while avoiding shrinkage of the volume, which was verified by visual inspection of the overlapped CT images and mask.

### Generation of the FE Model

The segmented vertebrae were exported as surface meshes (STL) and imported in the 3D modeling software Ansys^®^ SpaceClaim Release 20.2 (Ansys Inc., Canonsburg, PA, United States). Through a reverse engineering process (“SkinSurface” command), a 3D solid model of each vertebra was reconstructed. The surface at the bottom of the model, representative of the inferior endplate, was used to apply the boundary conditions.

**FIGURE 2:**
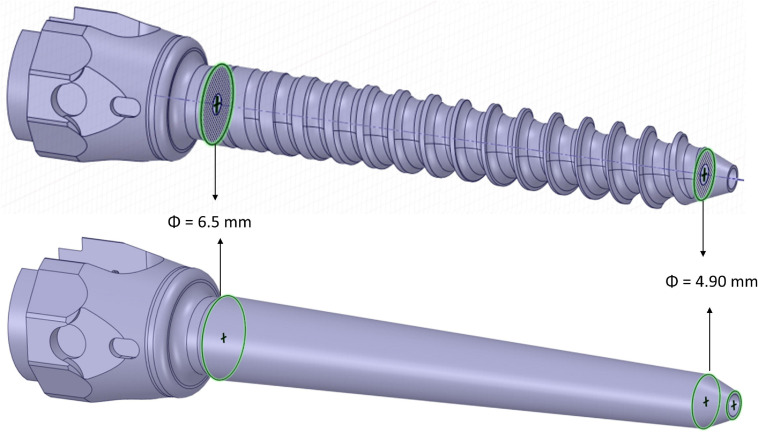
3D CAD models of simplified (bottom) and realistic (top) screws.

Afterward, the insertion of two pedicle screws (Aesculap^®^ S4^®^ Element MIS Monoaxial) was simulated. The realistic geometry of the implant was imported as STP file. Nine different sizes of pedicle screws available on the market were tested including D equal to 5.5, 6.5, or 7.5 mm and L equal to 45, 50, or 55 mm. Nine simplified screws with a smooth conic body without the thread were also generated to evaluate how the thread affected the loading distribution and deformation within the vertebra-screws construct. The solid model of the simplified screws was obtained from each of the nine realistic screws as following. The head of the screw until the end of the junction with the conic feature where the thread begins, and the last portion of the screw after the end of the thread were kept from the original realistic design. The two circular exposed sections were then connected with a conic surface (Figure 2). The realistic screws with the largest size (*D* = 7.5 mm, *L* = 50 mm) were virtually inserted at the pedicles by a Boolean subtraction. The insertion point was determined by following medical guidelines (Gertzbein and Robbins, 1990), which consist of finding the intersection point between a horizontal line passing through the transverse processes and a vertical line adjacent to the lateral border of the superior articular process. Screws were positioned parallel to the superior endplate, converging to the center of the vertebral body, keeping a distance of approximately 2.5 mm between the head of the screw and the superior articular processes (Figure 1). All other realistic and simplified screws were aligned to the position of the largest screws by registering their head, which were the same for every implant. Boolean subtraction from the original vertebra was applied for each pair of screws. In total eighteen models per vertebra were generated, nine with realistic geometries and nine with simplified geometries.

Each vertebra-screws construct was imported in Ansys^®^ Mechanical Enterprise Release 20.2 (Ansys Inc., Canonsburg, PA, United States) for meshing. The vertebra and the screws were meshed separately with tetrahedral quadratic elements (T10). For the vertebra, a uniform meshing algorithm was used so that the CT-scan grid was sampled uniformly during the definition of material properties of the bone. The element size was defined based on a mesh convergence study (see section “Generation of the FE Model”). A bonded contact was considered at the interface between the screws and the vertebra.

Bone was modeled as isotropic and heterogeneous material with Young’s modulus depending on the local BMD estimated from the CT images. In absence of an experimental densitometric calibration, the Hounsfield units were considered equal to BMD equivalent values (ρ_*QCT*_), using a scale factor to convert the physical units to g/cm^3^. This assumption was considered acceptable for the goal of this study, which is focused on the verification and sensitivity analysis of the models. From the BMD equivalent density, the apparent density (ρ_*App*_) was obtained through Eq. 1 (Schileo et al., 2008):

(1) $$\rho_{Q\,C\,T}=\rho_{A\,s\,h}=\rho_{A\,p\,p}\times 0.6\,\left(\frac{g}{c\,m^{3}}\right).$$

The Young’s modulus was then calculated using the density-elasticity experimental equation specific for thoraco-lumbar vertebrae (Eq. 2) (Morgan et al., 2003):

(2) $$E_{b\,o\,n\,e}=4730\,\rho_{A\,P\,P}^{1.56}$$

The Poisson’s ratio of the bone was set to *ν_*bone*_* = 0.3 (Wirtz et al., 2000). The values of *E*_*bone*_ were calculated and assigned for each element by using the Bonemat software (Taddei et al., 2007). The screw was considered isotropic and homogeneous with Young’s modulus and Poisson’s ratio of Titanium: *E*_*screw*_ = 102 GPa, *ν_*screw*_* = 0.36 (Niinomi and Boehlert, 2015).

The model was loaded with a quasi-static uniformly distributed force of 200 N (100 N per screw) applied to the head of the screw in a direction perpendicular to the longitudinal axis of the screw and perpendicular to the superior endplate, toward the caudal direction (Chen et al., 2003; Biswas et al., 2019; Figure 1). The force was equally distributed between the two surfaces of the head of the screw that would interact with the rod (50 N on each surface) (Figure 1). This load configuration aimed to represent the load exercised by the upper chest on the most inferior vertebra of a short-segment pedicle screw construct and transmitted by a rod that would be tightened in a direction perpendicular to the screw axis as estimated in an *in-vivo* study (Rohlmann et al., 1997). However, it should be noted that the model has a linear behavior and that the results of simulations were interpreted relative to the optimal configuration, therefore the magnitude of the load is not critical. In addition, the nodes of the inferior endplate of the vertebral body were fixed in all three directions (Chen et al., 2003). ANSYS^®^ Mechanical Enterprise Release 20.2 (Ansys Inc., Canonsburg, PA, United States) was used to solve the analysis. A workstation with processor model Intel(R) Xeon(R) CPU E5-2690 v3, 2.60 GHz was used. The analysis was run in parallel processing on 4 CPU Cores.

### Mesh Refinement Study

For each patient, the model configuration corresponding to the optimal screw size (*D* = 6.5 mm, *L* = 45 mm) as advised by surgeons was tested for verification purposes. A mesh convergence study was conducted to estimate the optimal mesh size. The element size was changed separately in the bone and pedicle screws. Six maximum element sizes were tested for the screws between 0.4 and 1.2 mm while keeping the element size in the bone constant and equal to 1 mm. A maximum element size larger than 1.2 mm resulted in an inaccurate discretization of the circular cavity of the screw’s body; the inferior boundary was considered at 0.4 mm based on the dimension of the smaller thread in the realistic screw (Table 2).

**TABLE 2:** Number of Elements and Degrees of Freedom per screw, averaged over the three patients, for six element sizes tested for the screws (the maximum element size is reported), in models with simplified or realistic screws; Total CPU time (time * number of CPU Cores) to solve models with simplified or realistic screws.

E-size screws (mm)	E-size vertebra (mm)	#Elements per simplified screw	#DOF per simplified screw	#Elements per realistic screw	#DOF per realistic screw	Total CPU time (s) simplified screw	Total CPU time (s) realistic screw
1.2	1	11,489	1.2 E+05	14,340	1.5 E+05	384	560
1.0	1	16,867	1.7 E+05	20,340	2.1 E+05	468	584
0.8	1	28,909	2.8 E+05	33,893	3.3 E+05	508	608
0.6	1	61,918	5.7 E+05	68,439	6.5 E+05	548	592
0.5	1	105,509	9.5 E+05	112,882	1.0 E+06	556	624
0.4	1	199,297	1.8 E+06	210,545	1.9 E+06	676	760

Moreover, maximum element sizes in the bone between 0.9 and 3 mm were tested for the finest mesh of the screw (0.4 mm) (Table 3). The lowest element size was to the voxel size of the CT-scan images of the three patients.

**TABLE 3:** Number of Elements and Degrees of Freedom in the vertebra, averaged over the three patients, for eight element sizes tested for the bone (the maximum element size is reported), in models with simplified or realistic screws; Total CPU time (time*number of CPU Cores) to solve models with simplified or realistic screws.

E-size vertebra (mm)	E-size screws (mm)	#Elements vertebra (simplified screw)	#DOF vertebra (simplified screw)	#Elements vertebra realistic screw	#DOF vertebra (realistic screw)	Total CPU time (s) (simplified screw)	Total CPU time (s) (realistic screw)
3.0	0.4	8,601	1.4 E+05	12,154	2.0 E+05	284	260
2.5	0.4	14,319	2.3 E+05	17,662	2.9 E+05	252	320
2.2	0.4	21,114	3.3 E+05	24,693	4.0 E+05	256	304
1.9	0.4	32,390	5.0 E+05	35,424	5.6 E+05	308	352
1.6	0.4	53,361	8.1 E+05	55,932	8.7 E+05	316	352
1.3	0.4	98,509	1.5 E+06	101,141	1.5 E+06	424	528
1.0	0.4	215,833	3.2 E+06	217,860	3.3 E+06	676	760
0.9	0.4	295,509	4.4 E+06	296,387	4.4 E+06	912	996

The computational time needed to solve the models with different element sizes is reported in Tables 2, 3. As the models were run in parallel computing, the total CPU time is calculated as the CPU time times the number of CPU cores. It should be noted that due to the heterogeneous properties of bone, the value of Young’s modulus in each element changes for different element sizes, making it impossible to uncouple the effect of mesh size from changes of material properties on the simulation outcomes. Therefore, the outcomes of the mesh refinement study should be interpreted by considering both changes in element size and material properties in the bone tissue.

The following metrics were considered for the different mesh sizes:

• The maximum total deflection (*d*_*max*_) of the head of the screw calculated as the magnitude of the displacement vector (nodal value).
• The peak Von Mises stress (*σ_*VM*_*) in the screws (nodal value) for the finest mesh. For the coarser meshes the *σ_*VM*_* was evaluated in the same coordinates, using the element shape functions to interpolate nodal values. Since the peak *σ_*VM*_* in the screws always occurred in a node on the external surface, for coarser meshes the coordinates of that node could fall outside the screw. To avoid this issue the outputs of the models were compared in a point within the volume of the screw at a distance equal to 0.05 mm from the point with peak *σ_*VM*_*.
• The peak Minimum Principal Strain (*ε_*p3*_*) in the bone (nodal value) for the finest mesh. For the coarser meshes the *ε_*p3*_* was evaluated in the same coordinates, using the element shape functions to interpolate nodal values. Some peaks were excluded from the analysis because their location was either close to the boundary conditions of the model, or in geometric sharp corners (for example close to the cuspid at the insertion point or close to the tip of screws), or in an area on the external surface of the vertebra potentially affected by segmentation problems (low values of Elastic modulus for the small elements of the finer mesh). In these cases, the next peak was considered.

### Influence of Screw Size and Geometry on Mechanical Properties of Screws-Vertebra Structure

Once the optimal mesh size was chosen for the bone and the screws, the influence of the diameter and length of screws on the stability of the simulated structure, for both the realistic and simplified models, was evaluated. The diameter and length of the left and right screws were changed simultaneously. The effect of changing the size of the screws was estimated with respect to the structural and local parameters estimated for the optimal screw size (*D* = 6.5 mm; *L* = 45 mm). The following parameters were calculated for the three patients:

• The maximum total deflection (*d*_*max*_) of the head of the screw calculated as the magnitude of the displacement vector (nodal value).
• The peak Von Mises stress (*σ_*VM*_*) in the screws (nodal value). Some peaks were excluded from the analysis because their location was close to the sharp corner of the bone geometry generated by the Boolean subtraction at the screw insertion point. This happened only for Patient #1, for a screw diameter of 5.5 mm. In this case the next peak, at a distance higher than five element sizes from the sharp corner, was considered in the analysis.
• The mean Minimum Principal Strain (*ε_*p3*_*) in the bone (nodal value). This value was calculated within a Region of Interest (ROI) defined at the screw-bone interface with a shape similar to the smooth conic body of simplified screws. The ROI was coaxial with the longitudinal axis of the screw and had a diameter equal to two times the diameter of the screw. Therefore, the dimensions of the ROI were scaled to each screw size. The same ROI was used for both simplified and realistic models.

For the patient characterized by the highest relative differences in *σ_*VM*_* in the screws and *ε_*p3*_* in the bone (Patient #2), the frequency plots for *ε_*p3*_* for three screw sizes (*D* = 7.5 mm and *L* = 50 mm; *D* = 6.5 mm and *L* = 45 mm; *D* = 5.5 mm and *L* = 40 mm) were compared for models with simplified and realistic screw geometries.

### Comparison Between Simplified and Realistic Screw Geometry

Linear regression analyses were performed between the predictions of *d*_*max*_ and *σ_*VM*_* from the models with simplified and realistic screw geometry. The Slope, Intercept and coefficient of determination (R^2^) were calculated for each linear regression.

## Results

### Mesh Refinement Study

**FIGURE 3:**
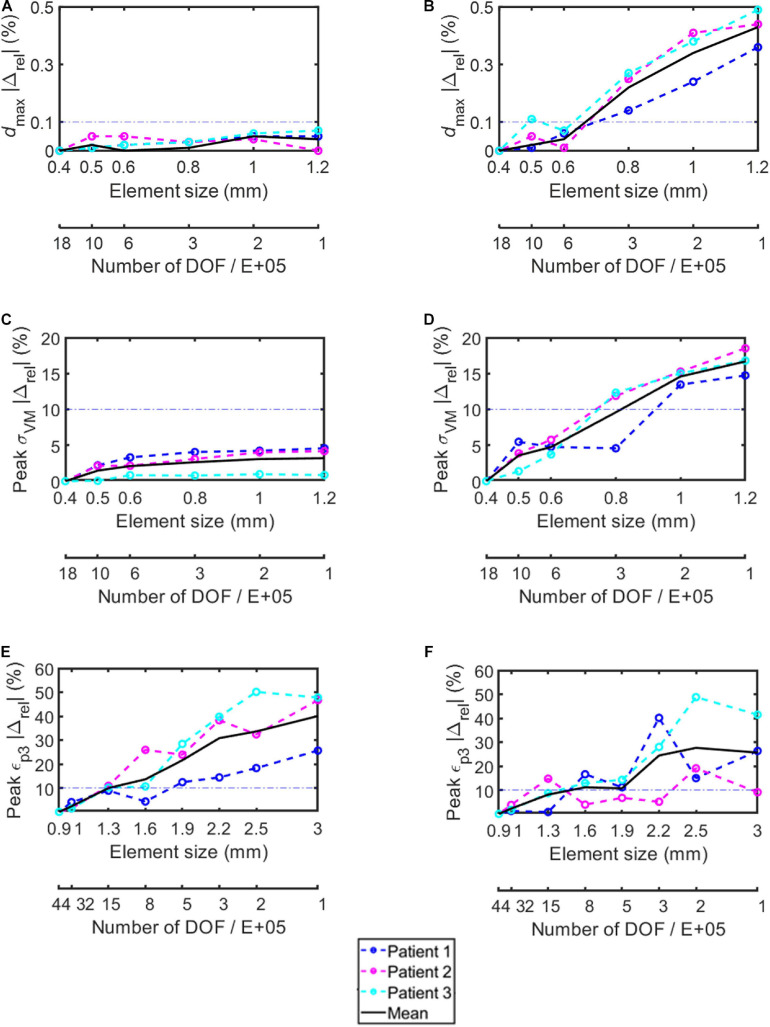
Percentage absolute difference with respect to the finer mesh in the simplified screws **(A,C,E)** and in the realistic screws **(B,D,F)** for maximum total deflection **(A,B)**, peak von Mises stress in the screw **(C,D)**, and peak minimum principal strain in the bone **(E,F)**. Dashed lines represent results for each subject, continuous black lines represent averaged values, dash-dotted lines represent the chosen 10% difference chosen as threshold.

The percentage absolute change with respect to the finer mesh in *d*_*max*_ for both simplified and realistic screw models was lower than 0.1% (screw) and 0.5% (bone) for each tested element size (Figures 3A,B).

**FIGURE 4:**
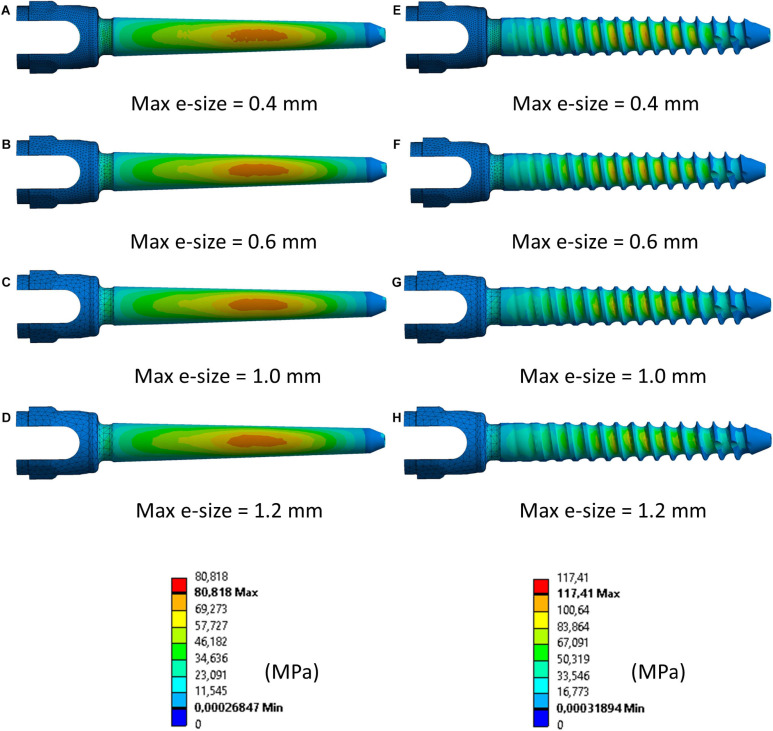
Distribution of von Mises stress in simplified **(A–D)** and realistic **(E–H)** left screws (Patient #2) for four different element sizes. Compressed fibers side (caudal view). The mesh was hidden in the main body of the screw to better visualize the stress distribution.

The percentage absolute change with respect to the finer mesh in peak *σ_*VM*_* was higher for the realistic screw compared to the simplified one (Figures 3C,D). In particular, while for the simplified model a percentage relative difference lower than 5% was observed for each tested element size, for the realistic case an element size of 0.6 mm allowed to achieve relative differences of approximately 5% or below. The *σ_*VM*_* distribution in the screws were similar for the models with different element size for both simplified and realistic screw geometry (Figure 4).

**FIGURE 5:**
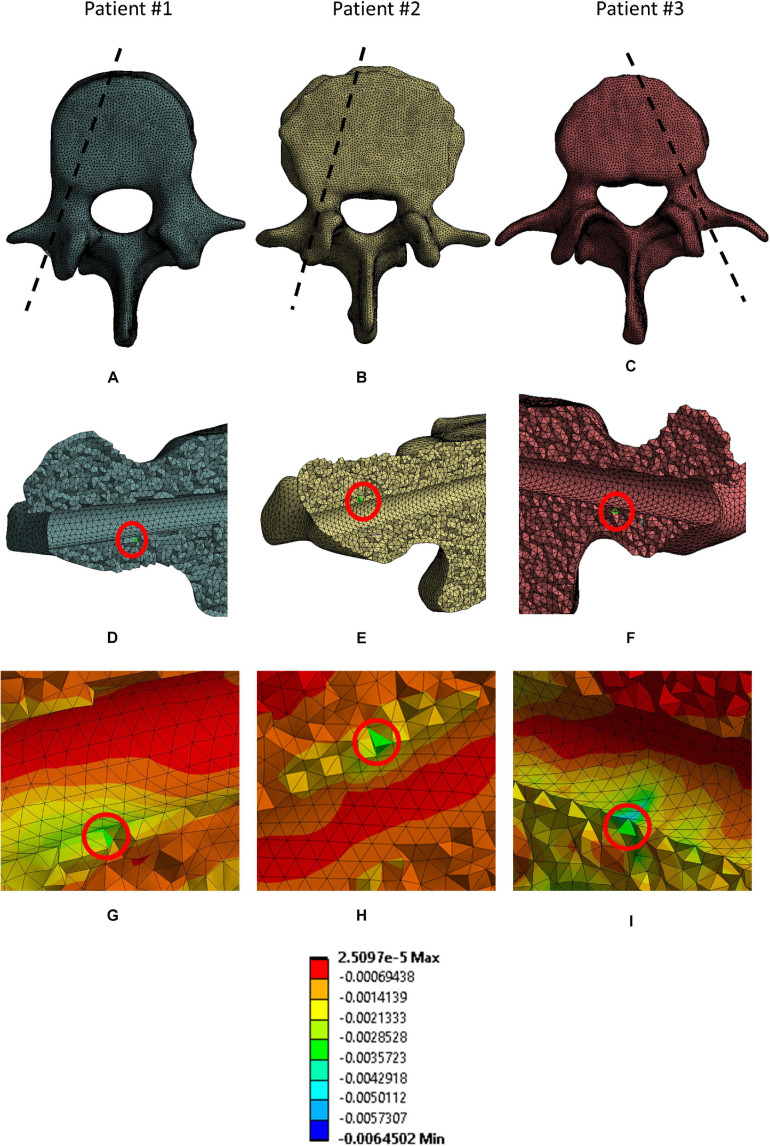
Location of elements where peak ε_*p3*_ in the bone were. For each patient the following views are reported: the projection of the sagittal section corresponding to the location of the elements in a cranial view **(A–C)**; the location of the peak (red circle) in a sagittal section for each patient **(D–F)**; a magnified view of the mesh in the area corresponding to the selected peaks **(G–I)**.

The peak *ε_*p3*_* values occurred at the interface between the bone and the left screw for Patient #1 and #2, and at the interface between the bone and the right screw for Patient #3 (Figure 5). The absolute percentage relative differences in peak *ε_*p3*_* were much higher than for the peak *σ_*VM*_*. For both simplified and realistic models, element size of 1 mm in the bone led to absolute percentage relative difference of approximately 5% or below for the three patients (Figures 3E,F).

For the following analyses, an element size of 0.4 mm was chosen in the screws because the computational time was not significantly affected (Tables 2, 3), and an element size of 1.0 mm was chosen in the bone.

### Effect of Size and Geometry of the Screw

The screw’s diameter had a more significant influence on *d*_*max*_ than the screw’s length in both simplified and realistic models, for both left and right screws (Table 4). Changes in length resulted in median values of percentage changes in *d*_*max*_ between 4 and 10%; whilst, changes in diameter resulted in median values of percentage changes in *d*_*max*_ between 28 and 36%. Similar changes were observed between right and left screws, for both simplified and realistic cases, and between simplified and realistic models, for both left and right screws. Very similar trends were found for the three patients. As expected, for a fixed length, the *d*_*max*_ increased for lower diameters; for a fixed diameter, the *d*_*max*_ decreased for longer screws.

**TABLE 4:** Percentage difference in maximum total deflection of the head of the screw, for simplified and realistic models, reported as median value and minimum and maximum values with respect to the nominal condition (*D* = 6.5 mm and *L* = 45 mm) over the three patients.

Effect of screw size and shape: Δ_*rel*_ (%) in *d*_*max*_				
Model–side	Length	Diameter		
		7.5 mm	6.5 mm	5.5 mm
0Simplified Left	40 mm	−10% (−9%, −10%)	4% (4%, −6%)	21% (18%, 24%)
	45 mm	−15% (−15%, −18%)	REF	18% (15%, 20%)
	50 mm	−19% (−19%, −23%)	−3% (−3%, −4%)	16% (13%, 17%)
Simplified Right	40 mm	−10% (−10%, −11%)	4% (4%, 5%)	20% (19%, 22%)
	45 mm	−16% (−15%, −17%)	REF	18% (17%, 19%)
	50 mm	−20% (−19%, −22%)	−3% (−2%, −3%)	16% (15%, 17%)
Realistic Left	40 mm	−8% (−8%, −9%)	5% (5%, 6%)	20% (18%, 23%)
	45 mm	−13% (−12%, 15%)	REF	17% (14%, 18%)
	50 mm	−16% (−15%, −18%)	−4% (−4%, −5%)	14% (11%, 14%)
Realistic Right	40 mm	−9% (−9%, −10%)	5% (4%, 6%)	21% (19%, 21%)
	45 mm	−13% (−13%, −14%)	REF	16% (15%, 17%)
	50 mm	−15% (−15%, −17%)	−4% (−3%, −4%)	13% (12%, 15%)

The diameter had higher impact on peak *σ_*VM*_* than the length for both simplified and realistic models (Table 5). In fact, changes in length resulted in median values of percentage changes in peak *σ_*VM*_* between 1 and 6%; instead, changes in diameter resulted in median values of percentage changes in peak *σ_*VM*_* between 6 and 27%. For both simplified and realistic models, similar percentage differences and trends were found between right and left screws. However, an asymmetry was found for Patient #1 in the models with realistic screws with *D* = 5.5 mm: for the three values of L, percentage differences in peak *σ_*VM*_* between 2 and 11% (left screws) and between 25 and 29% (right screws) were found. Since this patient had the largest pedicle among patients, models with screws with *D* = 5.5 mm were more sensitive to local changes in material properties. Generally, lower percentage differences in peak stress were found for the realistic screws compared to those obtained from simplified models. The percentage differences presented overall similar trends for the three patients. Also, the peak *σ_*VM*_* in the screw was higher in realistic models compared to those with simplified screws. For a fixed length, the *σ_*VM*_* increased for lower diameters; for a fixed diameter, the *σ_*VM*_* decreased for longer screws. However, in some cases with realistic screws, this behavior was not observed probably due to differences in local mechanical properties of bone adjacent to screws among models with different screw sizes.

**TABLE 5:** Percentage difference in peak Von Mises stress in the screws, for simplified and realistic models, reported with respect to the nominal condition (*D* = 6.5 mm and *L* = 45 mm) as median value and minimum and maximum values over the three patients.

Effect of screw size and shape: Δ_*rel*_ (%) of peak *σ_*VM*_*				
Model–side	Length	Diameter		
		7.5 mm	6.5 mm	5.5 mm
Simplified Left	40 mm	−10% (−8%, −13%)	1% (−4%, 4%)	15% (13%, 26%)
	45 mm	−13% (−10%, −13%)	REF	14% (2%, 19%)
	50 mm	−14% (−11%, −14%)	−1% (−1%, 0%)	9% (1%, 23%)
Simplified Right	40 mm	−12% (−9%, −14%)	0% (0%, 2%)	9% (6%, 37%)
	45 mm	−12% (−10%, −13%)	REF	10% (7%, 34%)
	50 mm	−14% (−14%, −16%)	−2% (−1%, −3%)	9% (5%, 35%)
Realistic Left	40 mm	−7% (−11%, 1%)	1% (−3%, 5%)	5% (−3%, 11%)
	45 mm	−7% (−10%, 1%)	REF	5% (0%, 7%)
	50 mm	−3% (−2%, −5%)	−4% (−2%, −4%)	3% (1%, 6%)
Realistic Right	40 mm	−4% (−9%, 0%)	3% (2%, 6%)	9% (5%, 27%)
	45 mm	−3% (−1%, −4%)	REF	5% (2%, 29%)
	50 mm	−3% (−3%, −6%)	−2% (−2%, −3%)	6% (4%, 25%)

For both simplified and realistic models, the diameter affected the mean *ε_*p3*_* more than the length (Table 6 and Figure 5). In fact, changes in diameter resulted in median values of percentage changes in mean *ε_*p3*_* between 30 and 47%, while changes in length resulted in median values of percentage changes in mean *ε_*p3*_* between 10 and 22%. For both simplified and realistic models, similar percentage differences and trends were found between right and left screws. Generally, similar percentage differences in mean *ε_*p3*_* were found for the models with realistic or simplified screws. Also, the mean *ε_*p3*_* in simplified models were similar to those with realistic screws. The percentage differences presented overall similar trends for the three patients. For a fixed length, the mean *ε_*p3*_* increased for lower diameters; for a fixed diameter, the mean *ε_*p3*_* decreased for longer screws.

**TABLE 6:** Percentage difference in mean Minimum principal strain in a ROI at the screw-vertebra interface, for simplified and realistic models, reported with respect to the nominal condition (D = 6.5 mm and L = 45 mm) as median value and minimum and maximum values over the three patients.

Effect of screw size and shape: Δ_*rel*_ (%) of mean *ε_*p3*_*				
Model–side	Length	Diameter		
		7.5 mm	6.5 mm	5.5 mm
Simplified Left	40 mm	−11% (−10%, −14%)	8% (7%, 11%)	33% (22%, 37%)
	45 mm	−22% (−17%, −22%)	REF	25% (17%, 25%)
	50 mm	−26% (−25%, −31%)	−7% (−5%, −10%)	15% (10%, 18%)
Simplified Right	40 mm	−10% (−7%, −13%)	8% (7%, 12%)	31% (26%, 31%)
	45 mm	−19% (−18%, −19%)	REF	20% (18%, 23%)
	50 mm	−25% (−23%, −27%)	−6% (−4%, −8%)	12% (11%, 17%)
Realistic Left	40 mm	−11% (−7%, −11%)	9% (8%, 10%)	34% (26%, 34%)
	45 mm	−17% (−17%, −17%)	REF	24% (20%, 24%)
	50 mm	−24% (−22%, −25%)	−6% (−6%, −10%)	12% (10%, 18%)
Realistic Right	40 mm	−12% (−6%, −13%)	9% (4%, 11%)	30% (30%, 31%)
	45 mm	−17% (−17%, −18%)	REF	19% (18%, 21%)
	50 mm	−22% (−21%, −23%)	−7% (−7%, −10%)	8% (8%, 14%)

### Comparison Between Simplified and Realistic Screw Geometry

**FIGURE 6:**
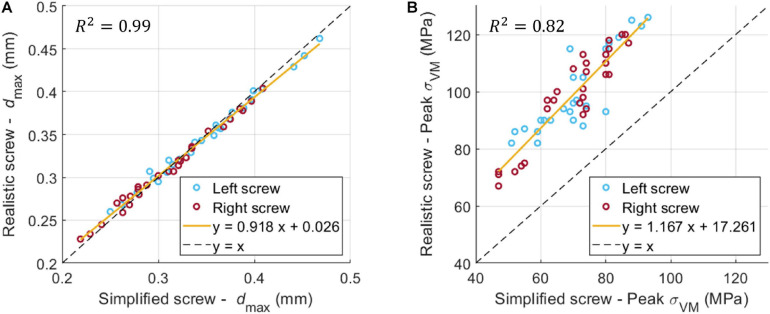
Linear correlation between d_*max*_ **(A)** and σ_*VM*_ **(B)** for the realistic and simplified models (data pooled for the three patients, two sides, nine sizes).

If data were pooled for the different patients, sizes and sides, the *d*_*max*_ calculated for models with realistic or simplified screws correlated very well (*R*^2^ = 0.99; Slope = 0.918, Intercept = 0.026 mm) (Figure 6A). A good correlation was also found between the peak *σ_*VM*_* calculated from the realistic and simplified models (*R*^2^ = 0.82) (Figure 6B). Nevertheless, the simplified models systematically underestimated the peak stress compared to the realistic ones (Slope = 1.2, Intercept ∼ 17 MPa).

**FIGURE 7:**
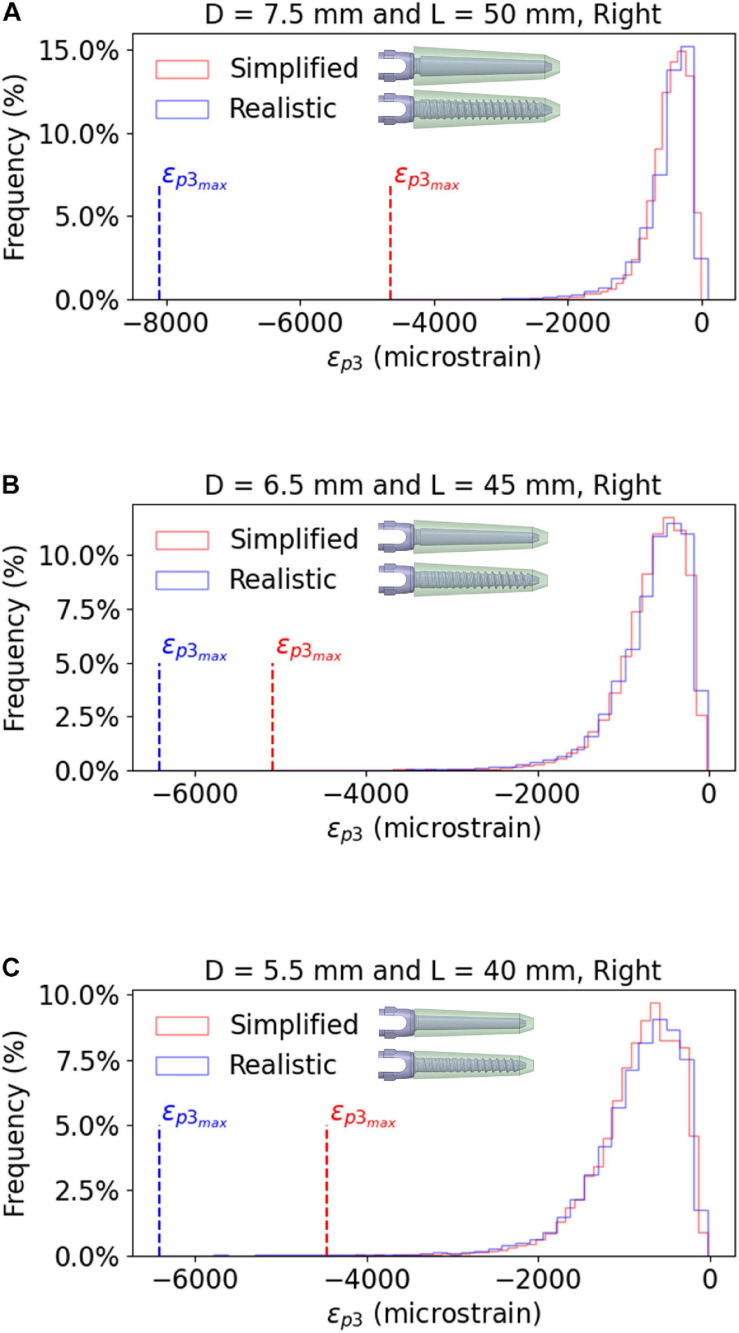
Frequency plots for the values of ε_*p3*_ within a bone ROI around the right screw, for realistic and simplified models. The data are reported for the largest screw size **(A)**, the optimal screw size **(B)** and the smallest screw size **(C)**, evaluated for Patient #2. Similar trends were observed for the other patients and the left screws.

The peak *ε_*p3*_* was highly influenced by the combination of screw geometry (simplified vs. realistic) and the distribution of Young’s modulus in the bone, whereas the distribution of values of *ε_*p3*_* within a ROI around the screw was similar for simplified and realistic design of screws, with only a localized increase of strain for a few elements in the realistic screw models (Figure 7).

## Discussion

This study aimed to generate and verify a subject-specific CT-based FE model of the human vertebra implanted with two pedicle screws. The model was then used to evaluate the effect of the size and geometry of the pedicle screws on the mechanical properties of the screws-vertebra structure.

Element sizes of 0.6 mm in the screw and 1.0 mm in the bone were associated to a relative difference of approximately 5% for both simplified and realistic models. Similarly, Costa et al. (2019) reported that element size of 1 mm was required for CT-based subject specific heterogeneous FE models of healthy non-instrumented vertebrae loaded in compression. Widmer et al. (2020) reported the results from a validation study for CT-based subject specific heterogeneous animal (bovine and porcine) vertebrae with realistic pedicle screws. They opted for smaller element sizes at the level of the screw cavity compared to the bone farer from the implant resulting in about 230,000 tetrahedral elements in the bone and 10,000 shell elements in the screw; however, no mesh refinement study was reported. Bianco et al. (2019) compared the fixation strength of realistic pedicle screws with different dimensions, bone engagement and entry point preparation under axial and non-axial forces, and chose an element size in the bone of approximately 0.3 mm around the screw thread and 1 mm in regions farer from the implant obtaining differences in results under 8% with respect to the finest mesh. It should be noted that little details are usually reported in the literature about the choice of the mesh size in models to simulate the biomechanics of vertebrae implanted with pedicle screws. This is critical as “verification” is one of the important steps to give credibility to the models for the assessment of the efficacy of medical devices (ASME, 2020).

As expected, in this study percentage relative differences in peak *σ_*VM*_* were higher in the realistic screws compared to the simplified ones. In fact, for the realistic screws different element sizes result in a more or a less accurate discretization of the thread features, which is not modeled in the simplified screw. It should be noted that the presence of the thread resulted in a 22–29% higher peak *σ_*VM*_* in realistic models compared to simplified ones for the baseline configuration (*D* = 6.5 mm, *L* = 45 mm). This was due to the fact that areas of concentration of stress occurred close to the thread, which may play a larger role compared to the diameter of the screw. However, the *σ_*VM*_* distribution over the screws was similar among the different mesh sizes for both realistic and simplified models, showing that the stress pattern is not much influenced by the element size.

The diameter of the screw had higher impact on the maximum displacement, on the peak *σ_*VM*_* in the screws and on the mean *ε_*p3*_* in the bone than the length of the screw. This shows that for mono-cortical screws the anchorage in the pedicle, which mainly consists of cortical bone, is more important compared to the anchorage within the vertebral body, which is mainly composed of trabecular bone. Therefore, finding the compromise between the largest diameter of the screws by avoiding iatrogenic fractures is crucial to provide a good anchorage on the cortical bone, which results in lower micromotions at the screw-vertebra interface and better distribution of stress, thus preventing post-operative complications. Our results are in line with most experimental and numerical studies in the literature, that showed the predominant effect of the diameter of the screws compared to their length (Zindrick et al., 1986; Chen et al., 2003; Cho et al., 2010; Matsukawa et al., 2016, 2020; Bianco et al., 2019; Biswas et al., 2019). Matsukawa et al. (2016) evaluated the effect of different screw sizes on fixation with a cortical bone trajectory, where screws are inserted pointing laterally in the transverse plane during superior screw angulation in the sagittal plane, and anchor only on cortical bone in the pedicle without the contribution of trabecular bone in the vertebral body (Santoni et al., 2009), by using an FE model including bone heterogeneities and realistic screw design. They found that some mechanical properties of the vertebra-screws construct were not significantly affected by increasing the diameter of screws. Even if our results for the impact of the diameter of the realistic screw seem to disagree with those by Matsukawa et al. (2016), this should be taken with caution as these differences may be due to different modeling techniques and different mechanical metrics used to evaluate the effect of the size of the screw. Matsukawa et al. (2020) investigated the effect of screw size on fixation in osteoporotic vertebrae by FE analysis. Their results showed that by increasing the diameter and the length of screws, the pullout strength and vertebral fixation strength increased; they also showed that the screw diameter had a more important effect than screw length on the vertebral fixation strength, similarly to the results of the present study. However, the modeling approach and boundary conditions of the two studies are substantially different so both outcomes are complementary.

Overall, the predictions of the simplified models correlated well with the predictions from the realistic ones, especially for the global structural properties (*d*_*max*_). This finding suggests that geometrical differences between the two designs of screws and local differences of material properties around the screw between the two models do not influence the overall stiffness of the model. Inzana et al. (2016) modeled a homogeneous cylindrical block of trabecular or cortical bone and compared a simplified cylindrical screw with a bonded interface and a realistic threaded screw with frictional contact with a pseudo-threaded screw with calibrated contact conditions. They found that the simplified model underestimated (70% difference, averaged value extracted from Figure 4 in that study) the displacement of the screw head with respect to the realistic case. They have also reported a similar overestimation of the global stiffness from the analysis of a model for the fixation of a proximal humerus fracture. In this study, it was found that the maximum deflection of the screw head was slightly higher in the simplified case, but a bonded interface was considered for both simplified and realistic models. The different results could be due to differences in material models, interfaces, geometries and applications between the two studies. Moreover, in this study the simplified models underestimated the peak *σ_*VM*_*, due to the lack of stress raisers considered in the realistic design. These differences could also be amplified by local heterogeneity in the Elastic modulus of bone elements. In fact, the distribution of Young’s modulus in the bone had a strong influence on the peak *ε_*p3*_*, whereas the distribution of strain around the screw-bone interface was similar for simplified and realistic models. This finding highlights the importance of the choice of modeling the screw’s geometry realistically or to use a simplified model depending on the application.

There are some limitations in this study. First, it is important to note that before this computational model can be used in the clinical setting, additional to the verification and sensitivity analysis of the model, a direct validation of this approach should be made with respect to measurements from *ex vivo* experiments. This study is the first step in the identification of the best approach to optimize the virtual assessment of pedicle fixation by accounting for realistic vertebral geometry and density distributions and by modeling the screw with a realistic or simplified geometry. Validation of the model against advanced time-lapsed mechanical testing, micro-CT imaging and digital volume correlation approaches (Dall’Ara et al., 2017) to measure the strain distribution in the bone tissue will follow in future studies. The screw-bone interface was modeled as perfect bonding. While this choice may lead to less realistic stress and strain patterns in the screw and in the bone, it simplifies the comparison between the models with realistic and simplified screws. Moreover, only the most inferior vertebra of a short-segment pedicle screw construct was modeled, excluding from the analysis the other features of the implanted spine unit. This choice was considered acceptable for this study that focused on vertical loads perpendicular to the axis of the screws. Nevertheless, in order to evaluate the effect of the screw size in physiological conditions, more complex geometry should be modeled. The insertion points of pedicle screws, as well as the orientation of the screw axes in the sagittal and transverse planes are important factors that influence the stress distribution on the screws and the bone. These two parameters should be considered in future parametric studies.

Finally, the effect of the size of the screw has been evaluated with nine discrete configurations instead of analyzing the possible range of parameters continuously with statistical methods. While this choice was driven by the configurations of screw size available in the market, a more general approach could have highlighted optimal combinations of diameter and length for the specific patients. In fact, the simplified design of the screw would allow to implement more easily a parametric model, and mesh morphing techniques could be applied to update the nodal positions to accommodate shape variations (Biancolini, 2017). This approach combined with reduced order modeling techniques could be used to accelerate the workflow and test several combinations of geometrical properties of the screw for a population of patients and to expand to non-linear analyses.

In conclusion, this study highlights the influence of size and geometry of screws on the biomechanics of a vertebra with two pedicle screws. In particular, the diameter of the screw should be optimized for each patient as it has a large impact on the stress in the screw. Moreover, modeling the screw with simplified geometry systematically underestimate the peak stress and should therefore be accounted for when interpreting the results from the FE analyses.

## Data Availability Statement

The raw data supporting the conclusions of this article will be made available by the authors by contacting the corresponding author. Results and example of the models are available at: https://bit.ly/37JflPc.

## Author Contributions

MS, TV, MR, and ED: research design. TV, CS, and TG: acquisition of data. MS and ED: analysis and interpretation of data. All authors drafted the manuscript and revised it critically, reviewed and agreed upon the final version of the manuscript.

## Conflict of Interest

MS and MR were employed by company Ansys (France). CS and TG were employed by company Aesculap (Germany). The remaining authors declare that the research was conducted in the absence of any commercial or financial relationships that could be construed as a potential conflict of interest.

## References

[B1] Abbeele M. V. den Valiadis J.-M. Lima L. V. P. C. Khalifé P. Rouch P. Skalli W. (2018). Contribution to FE modeling for intraoperative pedicle screw strength prediction. *Comput. Methods Biomech. Biomed. Eng.* 21 13–21. 10.1080/10255842.2017.1414200 29226718

[B2] ASME (2020). *Assessing Credibility of Computational Modeling through Verification and Validation: Application to Medical Devices – ASME.* Available online at: https://www.asme.org/codes-standards/find-codes-standards/v-v-40-assessing-credibility-computational-modeling-verification-validation-application-medical-devices (accessed October 26, 2020).

[B3] Bianco R.-J. Arnoux P.-J. Mac-Thiong J.-M. Aubin C.-E. (2019). Thoracic pedicle screw fixation under axial and perpendicular loadings: a comprehensive numerical analysis. *Clin. Biomech.* 68 190–196. 10.1016/j.clinbiomech.2019.06.010 31238188

[B4] Bianco R.-J. Arnoux P.-J. Wagnac E. Mac-Thiong J.-M. Aubin C. -É (2017). Minimizing pedicle screw pullout risks: a detailed biomechanical analysis of screw design and placement. *Clin. Spine Surg.* 30 E226–E232. 10.1097/BSD.0000000000000151 28323704

[B5] Biancolini M. E. (2017). “RBF mesh morphing,” in *Fast Radial Basis Functions for Engineering Applications*, ed. Biancolini M. E. (Cham: Springer International Publishing), 93–117. 10.1007/978-3-319-75011-8_6

[B6] Biswas J. K. Sahu T. P. Rana M. Roy S. Karmakar S. K. Majumder S. (2019). Design factors of lumbar pedicle screws under bending load: a finite element analysis. *Biocybern. Biomed. Eng.* 39 52–62. 10.1016/j.bbe.2018.10.003

[B7] Bredow J. Boese C. K. Werner C. M. L. Siewe J. Löhrer L. Zarghooni K. (2016). Predictive validity of preoperative CT scans and the risk of pedicle screw loosening in spinal surgery. *Arch. Orthop. Trauma Surg.* 136 1063–1067. 10.1007/s00402-016-2487-8 27312862

[B8] Campbell J. Q. Petrella A. J. (2016). Automated finite element modeling of the lumbar spine: using a statistical shape model to generate a virtual population of models. *J. Biomech.* 49 2593–2599. 10.1016/j.jbiomech.2016.05.013 27270207

[B9] Chen S.-I. Lin R.-M. Chang C.-H. (2003). Biomechanical investigation of pedicle screw–vertebrae complex: a finite element approach using bonded and contact interface conditions. *Med. Eng. Phys.* 25 275–282. 10.1016/S1350-4533(02)00219-9 12649011

[B10] Chevalier Y. Matsuura M. Krüger S. Fleege C. Rickert M. Rauschmann M. (2018). Micro-CT and micro-FE analysis of pedicle screw fixation under different loading conditions. *J. Biomech.* 70 204–211. 10.1016/j.jbiomech.2017.12.023 29336820

[B11] Cho W. Cho S. K. Wu C. (2010). The biomechanics of pedicle screw-based instrumentation. *J. Bone Joint Surg. Br.* 92 1061–1065. 10.1302/0301-620X.92B8.24237 20675747

[B12] Costa M. C. Eltes P. Lazary A. Varga P. P. Viceconti M. Dall’Ara E. (2019). Biomechanical assessment of vertebrae with lytic metastases with subject-specific finite element models. *J. Mech. Behav. Biomed. Mater.* 98 268–290. 10.1016/j.jmbbm.2019.06.027 31280054

[B13] Dall’Ara E. Peña-Fernández M. Palanca M. Giorgi M. Cristofolini L. Tozzi G. (2017). Precision of digital volume correlation approaches for strain analysis in bone imaged with micro-computed tomography at different dimensional levels. *Front. Mater.* 4:31. 10.3389/fmats.2017.00031

[B14] Elmasry S. Asfour S. Travascio F. (2017). Effectiveness of pedicle screw inclusion at the fracture level in short-segment fixation constructs for the treatment of thoracolumbar burst fractures: a computational biomechanics analysis. *Comput. Methods Biomech. Biomed. Engin.* 20 1412–1420. 10.1080/10255842.2017.1366995 28817960

[B15] Fedorov A. Beichel R. Kalpathy-Cramer J. Finet J. Fillion-Robin J.-C. Pujol S. (2012). 3D Slicer as an image computing platform for the Quantitative Imaging Network. *Magn. Reson. Imaging* 30 1323–1341. 10.1016/j.mri.2012.05.001 22770690 PMC3466397

[B16] Fior Markets (2020). *Global Pedicle Screw System Market by Product, Surgery Type, Indication, Application, Region, Industry Analysis, Size, Share, Growth, Trends, and Forecast 2018 to 2025 – Fior Markets.* Available online at: https://www.fiormarkets.com/report/global-pedicle-screw-system-market-by-product-polyaxial-375967.html (accessed October 26, 2020).

[B17] Gertzbein S. D. Robbins S. E. (1990). Accuracy of pedicular screw placement in vivo. *Spine* 15 11–14. 2326693 10.1097/00007632-199001000-00004

[B18] Li C. Zhou Y. Wang H. Liu J. Xiang L. (2014). Treatment of unstable thoracolumbar fractures through short segment pedicle screw fixation techniques using pedicle fixation at the level of the fracture: a finite element analysis. *PLoS One* 9:e99156. 10.1371/journal.pone.0099156 24914815 PMC4051693

[B19] Matsukawa K. Yato Y. Imabayashi H. (2020). Impact of screw diameter and length on pedicle screw fixation strength in osteoporotic vertebrae: a finite element analysis. *Asian Spine J.* 10.31616/asj.2020.0353 [Epub ahead of print]. 33355846 PMC8561163

[B20] Matsukawa K. Yato Y. Imabayashi H. Hosogane N. Abe Y. Asazuma T. (2016). Biomechanical evaluation of fixation strength among different sizes of pedicle screws using the cortical bone trajectory: what is the ideal screw size for optimal fixation? *Acta Neurochir. (Wien)* 158 465–471. 10.1007/s00701-016-2705-8 26769471

[B21] Molinari L. Falcinelli C. Gizzi A. Di Martino A. (2021). Effect of pedicle screw angles on the fracture risk of the human vertebra: a patient-specific computational model. *J. Mech. Behav. Biomed. Mater.* 116:104359. 10.1016/j.jmbbm.2021.104359 33548583

[B22] Morgan E. F. Bayraktar H. H. Keaveny T. M. (2003). Trabecular bone modulus–density relationships depend on anatomic site. *J. Biomech.* 36 897–904. 10.1016/S0021-9290(03)00071-X 12757797

[B23] Newcomb A. G. U. S. Baek S. Kelly B. P. Crawford N. R. (2017). Effect of screw position on load transfer in lumbar pedicle screws: a non-idealized finite element analysis. *Comput. Methods Biomech. Biomed. Engin.* 20 182–192. 10.1080/10255842.2016.1209187 27454197 PMC5386406

[B24] Niinomi M. Boehlert C. J. (2015). “Titanium alloys for biomedical applications,” in *Advances in Metallic Biomaterials: Tissues, Materials and Biological Reactions*. Springer Series in Biomaterials Science and Engineering, eds Niinomi M. Narushima T. Nakai M. (Berlin: Springer), 179–213. 10.1007/978-3-662-46836-4_8

[B25] Prud’homme M. Barrios C. Rouch P. Charles Y. P. Steib J.-P. Skalli W. (2015). Clinical outcomes and complications after pedicle-anchored dynamic or hybrid lumbar Spine stabilization: a systematic literature review. *J. Spinal Disord. Tech.* 28 E439–E448. 10.1097/BSD.0000000000000092 25093644

[B26] Qi W. Yan Y. Zhang Y. Lei W. Wang P. Hou J. (2011). Study of stress distribution in pedicle screws along a continuum of diameters: a three−dimensional finite element analysis. *Orthop. Surg.* 3 57–63. 10.1111/j.1757-7861.2010.00112.x 22009982 PMC6583207

[B27] Rajaee S. S. Bae H. W. Kanim L. E. A. Delamarter R. B. (2012). Spinal fusion in the United States: analysis of trends from 1998 to 2008. *Spine* 37 67–76. 10.1097/BRS.0b013e31820cccfb 21311399

[B28] Rohlmann A. Bergmann G. Graichen F. (1997). Loads on an internal spinal fixation device during walking. *J. Biomech.* 30 41–47. 10.1016/S0021-9290(96)00103-0 8970923

[B29] Santoni B. G. Hynes R. A. McGilvray K. C. Rodriguez-Canessa G. Lyons A. S. Henson M. A. W. (2009). Cortical bone trajectory for lumbar pedicle screws. *Spine J.* 9 366–373. 10.1016/j.spinee.2008.07.008 18790684

[B30] Schileo E. Dall’Ara E. Taddei F. Malandrino A. Schotkamp T. Baleani M. (2008). An accurate estimation of bone density improves the accuracy of subject-specific finite element models. *J. Biomech.* 41 2483–2491. 10.1016/j.jbiomech.2008.05.017 18606417

[B31] Su Y. Wang X. Ren D. Liu Y. Liu S. Wang P. (2018). A finite element study on posterior short segment fixation combined with unilateral fixation using pedicle screws for stable thoracolumbar fracture. *Medicine (Baltimore)* 97:e12046. 10.1097/MD.0000000000012046 30142856 PMC6112892

[B32] Taddei F. Schileo E. Helgason B. Cristofolini L. Viceconti M. (2007). The material mapping strategy influences the accuracy of CT-based finite element models of bones: an evaluation against experimental measurements. *Med. Eng. Phys.* 29 973–979. 10.1016/j.medengphy.2006.10.014 17169598

[B33] Verma K. Boniello A. Rihn J. (2016). Emerging techniques for posterior fixation of the lumbar Spine. *J. Am. Acad. Orthop. Surg.* 24 357–364. 10.5435/JAAOS-D-14-00378 27077477

[B34] Wang W. Pei B. Pei Y. Shi Z. Kong C. Wu X. (2019). Biomechanical effects of posterior pedicle fixation techniques on the adjacent segment for the treatment of thoracolumbar burst fractures: a biomechanical analysis. *Comput. Methods Biomech. Biomed. Engin.* 22 1083–1092. 10.1080/10255842.2019.1631286 31225742

[B35] Widmer J. Fasser M.-R. Croci E. Spirig J. Snedeker J. G. Farshad M. (2020). Individualized prediction of pedicle screw fixation strength with a finite element model. *Comput. Methods Biomech. Biomed. Engin.* 23 155–167. 10.1080/10255842.2019.1709173 31910656

[B36] Wirtz D. C. Schiffers N. Pandorf T. Radermacher K. Weichert D. Forst R. (2000). Critical evaluation of known bone material properties to realize anisotropic FE-simulation of the proximal femur. *J. Biomech.* 33 1325–1330. 10.1016/S0021-9290(00)00069-5 10899344

[B37] Zindrick M. R. Wiltse L. L. Widell E. H. Thomas J. C. Holland W. R. Field B. T. (1986). A biomechanical study of intrapeduncular screw fixation in the lumbosacral spine. *Clin. Orthop. Relat. Res.* 203 99–112. 3956001

